# Implications of Resveratrol on Glucose Uptake and Metabolism

**DOI:** 10.3390/molecules22030398

**Published:** 2017-03-07

**Authors:** David León, Elena Uribe, Angara Zambrano, Mónica Salas

**Affiliations:** 1 Instituto de Bioquímica y Microbiología, Facultad de Ciencias, Universidad Austral de Chile, Valdivia 509000, Chile; davidleonn90@gmail.com; 2 Departamento de Bioquímica y Biología Molecular, Facultad de Ciencias Biológicas, Universidad de Concepción, Concepción 4030000, Chile; auribe@udec.cl; 3 Center for Interdisciplinary Studies on the Nervous System (CISNe), Universidad Austral de Chile, Valdivia 509000, Chile

**Keywords:** resveratrol, polyphenols, glucose transport and metabolism, cancer, diabetes, signal transduction

## Abstract

Resveratrol—a polyphenol of natural origin—has been the object of massive research in the past decade because of its potential use in cancer therapy. However, resveratrol has shown an extensive range of cellular targets and effects, which hinders the use of the molecule for medical applications including cancer and type 2 diabetes. Here, we review the latest advances in understanding how resveratrol modulates glucose uptake, regulates cellular metabolism, and how this may be useful to improve current therapies. We discuss challenges and findings regarding the inhibition of glucose uptake by resveratrol and other polyphenols of similar chemical structure. We review alternatives that can be exploited to improve cancer therapies, including the use of other polyphenols, or the combination of resveratrol with other molecules and their impact on glucose homeostasis in cancer and diabetes.

## 1. Introduction

Resveratrol (RSV) is a natural polyphenol found in grapes and other vegetable products of human consumption that drew the attention of scientists in 1997 when a study published by Jang and colleagues revealed its antioxidant and chemopreventive properties [1]. Many studies consider RSV a “magical molecule” due to its multiple targets in cells, including processes and signaling pathways concerning inflammation [2], reduction of oxidative stress [3], apoptosis [4,5], and anticancer effects [6,7]. It is because of these pleiotropic effects that scientists consider RSV to have potential as an anticancer drug, and efforts have been made to thoroughly understand its mechanisms of action.

With regards to cancer, it is known that malignant cells have a high dependency on the glycolytic pathway to supply their need for energy and metabolic intermediates to the point that they shift their main adenosine triphosphate (ATP)-producing process from oxidative phosphorylation to glucose fermentation, even in aerobic conditions [8]. Since this metabolic shift produces less ATP per glucose molecule, the demand for glucose in these cells is higher than normal cells. In order to keep a constant supply of glucose, cancer cells often overexpress the glucose transporter 1 (GLUT1) facilitative carrier [9,10,11,12]. The difference in energy metabolism between normal and cancer cells constitutes a biochemical basis for targeting glucose metabolism as a therapeutic approach in cancer treatment. A promising strategy for cancer treatment is the inhibition of glucose transporters in neoplastic cells that aims to generate an energy deprivation state that can facilitate the effect of other anticancer therapies [13,14].

One of the biggest challenges for RSV in therapy is its poor bioavailability. Due to its rapid phase II metabolism in liver and intestine [15,16], the bioavailability of ingested and intravenous doses of RSV is unable to achieve pharmacologically active concentrations in plasma [17]. In order to overcome this challenge, the use of natural or synthetic analogs that have better bioavailability or more potency than RSV, as well as combinations of drugs that result in a synergistic effect or in improvements on its bioavailability are promising strategies, as in the case of quercetin and other flavonoids [18]. This last method is very attractive for anticancer drug therapy because the combination of drugs might result in the use of lesser doses of individual compounds, leading to better pharmacological action due to additive or synergistic effects, and less collateral effects on the organism [19,20].

The aim of this review is to collect and present the most recent evidence in the literature (last 10 years) regarding RSV and its effects on cell uptake and metabolism of glucose in physiological conditions as well as in cancer and type 2 diabetes.

## 2. Effects of Resveratrol in Physiological Conditions

Before exploring the action of RSV in the abovementioned pathologies, it is of interest to know if RSV has some protective effect and can elicit a significant response on the glucose handling in healthy individuals. Kang and colleagues performed a study to test the effects of RSV on the response to insulin in skeletal muscle, liver, and adipose tissue extracted from healthy and diabetic rodents, concluding that the polyphenol enhances the effect of insulin only in insulin-resistant rodents [21]. In accordance with these results, clinical trials performed in obese [22] and healthy patients confirmed that RSV is not able to modulate the response to insulin in non-diabetic individuals. Finally, a meta-analysis by Liu et al. [23] of eleven clinical trial studies confirmed that RSV only improves insulin response in diabetic individuals. The results from these mice and human experiments heavily suggest that RSV is not able to modulate the response to insulin in healthy patients; hence, its role as a prophylactic agent against type 2 diabetes cannot be assured.

## 3. Effects of Resveratrol in Type 2 Diabetes Mellitus

The effects of RSV on glucose uptake and metabolism have been thoroughly studied in diabetic animal models, as well as in clinical trials. Type 2 diabetes arises from a resistance to insulin signaling in its main target tissues: skeletal muscle, liver, and adipose tissue, which results in a disruption of body glucose homeostasis that increases blood glucose levels, leading to a series of health complications including renal disease and higher risk of heart disease and stroke [24,25].

In accordance with the experimental evidence shown previously, RSV has been thoroughly tested for its antidiabetic properties. In contrast to the lack of a significant response observed in healthy mice, RSV improved the response of obese insulin-resistant mice to insulin in the same study [26], measured as an increase in a serine-threonine protein kinase that encodes protein kinase B (Akt) phosphorylation in adipose tissue and liver. In muscle, RSV treatment did not increase insulin-mediated Akt phosphorylation, but it increased the phosphorylation (and activation) of the α-subunit of adenosine monophosphate (AMP)-activated kinase (AMPK). This kinase has a central role in the control of the cellular energy state, regulating protein synthesis and lipid and glucose metabolism [27], and it also increases glucose uptake by stimulating the translocation of GLUT4 vesicles from the cytosol to the plasma membrane in skeletal muscle and adipose tissue [28,29,30]. Further research on the relationship between the activation of AMPK by RSV and the hypoglycemic effect observed in rodents could give us a better understanding of the polyphenol´s antidiabetic effects.

According to this, Breen and colleagues investigated the effect of RSV on mice L6 myotubes, and confirmed that the increase in glucose uptake observed in the treatment with RSV relies on the gene that encodes a widely expressed nicotinamide/dependent protein deacetylase (*SIRT*)-dependent activation of AMPK [31]. Interestingly, their results in this model also suggest that RSV does not stimulate the translocation of GLUT4 to the plasma membrane nor does it increase its expression, but it might increase the intrinsic activity of the transporter. An in vivo study also supports the idea that RSV increases glucose uptake in mice skeletal muscle without stimulating GLUT4 expression after 16 months of treatment [32]. In general, insulin stimulates glucose uptake by translocation of glucose transporter GLUT-4 from intracellular pool to the caveolar membrane system. Caveolin-3 (CAV-3)—a member of the caveolin family—is involved in insulin-stimulated glucose uptake. In other models of muscle cells, it has been demonstrated that RSV also elevates insulin-dependent glucose uptake by enhancing GLUT4 translocation to the plasma membrane. Interestingly, RSV increased CAV-3 protein expression, which contributed to this translocation [33].

Furthermore, clinical studies in humans have revealed the beneficial effects of RSV on type 2 diabetes in humans, such as improvements in insulin resistance and reduction of oxidative stress [34], blood urea, hemoglobin glycosylation, and total cholesterol [35], and both fasting and post-meal blood glucose [36]. A clinical study on a cohort of 10 male diabetic patients revealed that a 12-week RSV treatment induced the upregulation of GLUT4 by AMPK phosphorylation in skeletal muscle [37], revealing one of the putative molecular targets that may induce the observed antidiabetic effects of RSV. These results, together with the aforementioned animal studies, represent a solid ground to better understand the effects of RSV on type 2 diabetes and to provide a base for further research on the therapeutic effects of this polyphenol.

## 4. Resveratrol as an Inhibitor of Glucose Uptake and Metabolism in Cancer

As stated above, malignant cells supply their demand of glucose by overexpressing transporters such as GLUT1 [38,39,40,41]. Since the anticancer properties of RSV have been previously demonstrated, it makes sense to investigate if the anticancer properties of RSV can be related to an effect of the polyphenol on the glucose uptake and metabolism of cancer cells.

A study published by Kueck and colleagues assessing the effect of RSV on the viability and glucose uptake on five human ovarian cancer cell lines found that treatments of up to 8 hours were able to reduce glucose uptake, lactate production, Akt, and mammalian target of rapamycin (mTOR) signaling and cell viability in a dose- and time-dependent manner [42,43]. The authors concluded that the energy deprivation state caused by RSV in ovarian cancer cells induced autophagy-mediated cell death. In the same context, Gwak and collaborators [44] first demonstrated that RSV inhibits glucose uptake in four ovarian cancer cells through the interruption of plasma membrane trafficking of the GLUT1 in an Akt/mTOR dependent manner. Both studies can be related to the extent and importance of the Akt/mTOR pathway for glucose uptake in ovarian cancer cells, and its potential as a target for pharmacological inhibition by RSV or other drugs in anticancer therapy.

Regarding the regulation of the enzymes involved in glucose metabolism, Gómez and collaborators described for the first time the ability of RSV to directly inhibit the function of phosphofructokinase 1 (PFK-1) in MCF-7 human breast cancer cells and purified enzyme extracts [45]. They correlated this effect with an observed decrease in glucose uptake and lactate production in these cells, highlighting the importance of the regulatory role of PFK-1 in glycolysis.

Expression of pyruvate kinase M2 (PKM2) switches metabolism to promote proliferation of cancer cells. Interestingly, RSV down-regulates PKM2 expression by inhibiting mTOR signaling, inducing a decrease in glucose uptake, lactate production, and reducing anabolic pathways in various cancer cell lines [46].

There are other cell factors that influence glucose uptake besides transporter expression and the activation of enzymes and signaling pathways. A recent study by Kyung and colleagues suggests that the decrease of glucose uptake by RSV observed in mouse Lewis lung carcinoma cells (3LL) depends on its antioxidant properties. The authors theorized that the presence of reactive oxygen species (ROS) and activation of hypoxia inducible factor (HIF-1α) stimulate glucose uptake in this cell model [47]. The aforementioned studies evaluate the effect of RSV on glucose uptake using experimental transport times higher than 30 min. It has been demonstrated that in order to discriminate between transport and glucose accumulation by phosphorylation into glucose-6-phosphate it is necessary to carry transport experiments using very short times, usually less than 1 min [48]. According to this rationale, we observed the direct inhibitory effect of RSV on the GLUT1 carrier in two human leukemic cell lines (U-937, HL-60), and in human erythrocytes at both short and long times, indicating that RSV can inhibit GLUT1-mediated transport and hekoxinase-mediated trapping of glucose. Our results show for the first time a direct interaction of RSV with GLUT1, binding to its internal face and behaving as a noncompetitive inhibitor [49].

We consider that further research on the molecular interaction between RSV and its targets on the cell like GLUT1 (whose crystallographic structure is now available in the literature [50]) is necessary to understand the molecular bases of its pharmacological effects. This knowledge can also be used to predict the mechanism of action of natural or synthetic compounds of similar structure and help to identify new potential natural drugs that may have a similar or stronger effect than RSV.

## 5. Signal Transduction Induced by Resveratrol on Glucose Metabolism

RSV has multiple molecular targets at the cellular level, many of which are related to cancer and type 2 diabetes. Specifically, RSV induces intracellular signal transduction pathways, which causes changes in the gene expression pattern of the target cells [51]. Originally it was discovered as a cyclooxygenase inhibitor, has also been identified as an activator of SIRT1 (inhibitor of cyclic AMP phosphodiesterases), and it is related to many other signaling pathways [52,53].

In mammals, sirtuins compose a family of several proteins, from SIRT1 to SIRT7. SIRT1 has important effects of caloric restriction and lifespan extension [54,55]. Many of the metabolic pathways that are influenced by SIRT1 are also altered in tumor development. SIRT1 is able to regulate oncogenic factors, controlling many aspects of metabolism [56]. Resveratrol is the most potent activator of SIRT1, so RSV triggers the deacetylation of many metabolic transcriptional regulators in vivo [57]. Proliferator-activated receptor-gamma coactivator-1 (PGC-1) is one well-characterized target of SIRT1 that acts as an essential regulator of mitochondrial biogenesis [58]. Upon deacetylation by SIRT1, PGC-1 increases its activity, controlling mitochondrial gene expression [59].

On the other hand, SIRT1 also regulates the FOXO (Forkhead O box) family of transcription factors [60]. FOXO1—a member of this family—is an important regulator of the insulin signaling pathway which has an inhibitory role in glucose uptake and utilization. Phosphorylation of FOXO1 is an important controlling metabolism, specifically in regulating the gene expression of several genes, such as pyruvate dehydrogenase lipoamide kinase 4 (PDK4) and pyruvate dehydrogenase (PDH). It has been proven that RSV is able to modulate the SIRT1–FOXO1 signaling axis [61].

Another important protein regulated by resveratrol is AMP-activated kinase [62,63]. AMPK is an ubiquitously expressed metabolic sensor in several species. AMPK is a nutrient-sensing enzyme that is activated by an increase in the AMP/ATP ratio, which reflects a decrease in the energy status of the cell [64]. Although AMPK is controlled by the AMP/ATP ratio, there are other proteins that play a major role in the AMPK pathway. Two kinases are able to phosphorylate AMPK on the T172 residue necessary for its activation: LKB1 (Liver kinase B1) and calcium/calmodulin-dependent kinase kinase-β (CaMKKβ) [64]. However, there is scarce information about the LKB1 or CaMKKβ induction by RSV. Interestingly, AMPK and SIRT1 share a number of targets, including the FOXO transcription factors, suggesting a clear interaction between these signaling systems.

Mammalian target of rapamycin is one of the downstream targets of AMPK, and has an important role as an intracellular nutrient sensor to control protein synthesis, cell growth, proliferation, and metabolism [65,66]. RSV inhibits mTOR signaling via a SIRT1-independent mechanism, repressing protein synthesis [67].

In general, mTOR signaling is regulated by different signaling pathways, such as the mitogen-activated protein kinase pathway, the phosphatidylinositol 3-kinase pathway, and the AMP-activated protein kinase pathway [68,69,70,71]. Phosphatidylinositol 3-kinase is associated with the activation of Akt, another target of RSV. In fact, Akt is activated by its binding with membrane phospholipids, and it phosphorylates two downstream kinases: PDK1 (phosphoinositide-dependent kinase 1), and mTOR complex. Akt activation modulates several important functions for cellular physiology, such as cell survival, cell growth, and cell metabolism [72]. RSV prevented the activation of Akt by a several different stimuli [73,74]. It has also been demonstrated that RSV interrupts intracellular GLUT1 trafficking to the plasma membrane in ovarian cancer cells by the inhibition of Akt activity, as confirmed by the action of the Akt inhibitors (LY294002 and Akt inhibitor IV) [44]. This is another possible mechanism of the crosstalk between signaling pathways induced by RSV and its effect on glucose uptake.

## 6. Other Natural Polyphenols and Their Effect on Cancer Cells Glucose Uptake

Thanks to the explosive increase of published studies on RSV and its anticancer properties, scientists have started to take interest in other natural compounds in the search for new anticancer drugs. We will now review some of the most recent studies on natural polyphenols and RSV analogs, indicating some of the general knowledge on these drugs and then focusing on the scientific evidence that demonstrates their effect on glucose uptake and metabolism in cancer cells.

Kaempferol is a flavonoid whose cytotoxic effects have been demonstrated in several cancer models, including leukemia [75], lung cancer [76], gastric cancer [77], and bladder cancer [78]. A recent study by Azevedo et al. reported that kaempferol is able to inhibit glucose uptake in the MCF-7 breast cancer cell line with an IC_50_ of 4.0 μM [79]—a value at least 10 times lower than the reported value for RSV (67.2 μM). The kinetic analysis of the inhibition of 2-DOG (2-deoxy-d-glucose) demonstrated that kaempferol behaves as a mixed inhibitor because it increased both the Michaelis-Menten (*K*_M_) and maximum reaction velocity (*V*_max_) constant values of transport in these cells. It draws our attention that a mixed inhibitor should display a decrease of the *V*_max_ instead of an increase, so further studies are needed to properly assess the behavior of this compound.

Another polyphenol whose pharmacologic properties have been thoroughly studied is curcumin (diferuloylmethane), a natural compound extracted from the rhizome of turmeric (*Curcuma longa*); it possesses multiple targets in the cell that are crucial to the development of cancer [80,81,82,83,84]. Recently, a report by Gunnink and colleagues [85] demonstrated for the first time that curcumin can inhibit the glucose uptake in the L929 mouse fibroblast cell line by binding to a site overlapping the cytochalasin B binding site of the GLUT1 glucose carrier, acting as a mixed inhibitor. They also hypothesize that the inhibitory effects observed in the intestine due to curcumin could be caused by an inhibition of GLUT2-mediated glucose uptake.

Finally, nordihydroguaieretic acid (NDGA) is a polyphenol extracted from the creosote bush *Larrea tridentata*. Extracts of this plant have been used in folk medicine to treat different diseases including biliary and kidney stones, inflammation, arthritis, and sexually transmitted diseases [86], and the antioxidant and antitumor properties of NDGA have been thoroughly studied [87,88,89,90,91]. Due to its structural similarities with RSV, we decided to study NDGA in order to find an effect on glucose transport in cancer cells. We demonstrated that NDGA inhibits glucose uptake in a noncompetitive way in the HL-60 and U-937 human leukemic cell lines, both in short-time assays (40 s) and long-time (40 min). Transport assays in human red blood cells suggest that this polyphenol directly interacts with the GLUT1 carrier binding to a region different to the substrate binding site.

## 7. Combinatory Studies of Resveratrol and Other Drugs Related to Glucose Metabolism

An in vivo approach using the combination of curcumin and RSV in lung carcinogenesis reported a significant decrease in cancer cell proliferation, glucose uptake and metabolism, and induction of apoptosis via higher expression and phosphorylation of p53, which eventually led to a higher activity of caspase enzymes, concluding that the combination of both phytochemicals had a synergistic effect on cell proliferation but not on glucose uptake, since RSV alone was enough to achieve the same effect than the combination [92]. These results could help us better understand the differences in the molecular targets and mechanisms of action of two pleiotropic drugs that have similar cellular effects.

Another study that assessed the effect of a combined treatment of RSV and metformin on type 2 diabetes in mice found that the combination was able to significantly improve insulin and glucose resistance when compared to the untreated control, while neither RSV nor metformin alone were able to improve it. The study suggests that the observed improvement in glucose homeostasis caused by the drug combination may involve an enhancement of insulin signaling in adipose tissue and skeletal muscle, as observed by higher Akt phosphorylation in this tissue [93].

## 8. Conclusions

The studies reviewed in this article show the increasing interest of the scientific community in identifying the molecular and cellular mechanisms of action of RSV and other phytochemicals with anticancer properties. Despite the growing body of studies performed in vitro, in animal models, and in humans, the studies on the effects of RSV on glucose transport and metabolism in both cancer and type 2 diabetes are mostly limited to signaling pathways, and little is known about its putative effects on downstream elements like enzymes and transporters. In addition, there is no clear demonstration of the protective effects in healthy individuals against cancer or diabetes. We have presented some of the studies that delve into this yet unexplored territory, bringing new molecular targets of natural polyphenols and their combinations that could mean promising results for the mentioned pathologies.

One of the controversies that arose from the research of the therapeutic properties of RSV in cancer and type 2 diabetes is the opposite effects observed on glucose uptake in both disease models. RSV is able to block glucose uptake in cancer cells, affecting the survival, while in skeletal muscle and adipose tissue, it enhances insulin-stimulated glucose uptake. However, there are reports showing that RSV seems to have different effects depending on its dose (hormesis). Acute exposure to low doses (<10 µM) of RSV stimulates glucose uptake in skeletal muscle and adipose tissue in both human and rodent models, while higher doses (>100 μM) inhibit glucose uptake by impairing insulin action [94,95]. These results challenge the paradigm of the antidiabetic effects of RSV, but before a new point of view can arise, we must first clarify how different RSV concentrations and times of exposure elicit different responses on its target cells. In cancer cells, the main glucose transporter is GLUT1, a facilitative carrier which expression related to the activation of the mTOR pathway—a known target of inhibition by RSV. On the other hand, the activity and expression of GLUT4—the main glucose transporter in insulin-responding tissues—depends on the signaling pathways activated by insulin, like AMPK.

We hypothesize that the polyphenols like RSV—which share structural similarities and inhibit glucose uptake—could generate inhibition of cell proliferation on malignant cells, finally inducing cellular death by a mechanism that is still unknown.

There is still a long way before we can assess the clinical efficacy and safety of RSV, because its poor bioavailability constitutes one of the major challenges. The clinical studies and drug delivery strategies that are being researched promise a future in which we will finally know if this phytochemical lives up to its reputation, or if other natural polyphenols rise as better alternatives in the treatment of these diseases.
